# Barnacle-like lesions in the gastric mucosa: Clinicopathological study of a novel endoscopic finding

**DOI:** 10.1055/a-2645-7506

**Published:** 2025-07-29

**Authors:** Aya Sunago, Takahisa Murao, Ken Haruma, Maki Ayaki, Noriaki Manabe, Minoru Fujita, Takashi Akiyama, Mitsuhiko Suehiro, Hirofumi Kawamoto, Kazuhiko Inoue, Katsuhiro Mabe, Eiichiro Kanda, Tomoari Kamada

**Affiliations:** 1 Health Care Medicine, Kawasaki Medical School General Medical Center, Okayama, Japan; 2 12864 General Internal Medicine 2, Kawasaki Medical School, Kurashiki, Japan; 3 Gastroenterology and Hepatology, HITO Medical Center, Shikokuchuou, Japan; 4 12864 Division of Endoscopy and Ultrasonography, Department of Clinical Pathology and Laboratory Medicine, Kawasaki Medical School, Kurashiki, Japan; 5 Pathology, Kawasaki Medical School General Medical Center, Okayama, Japan; 6 Health Care Medicine, Junpukai Health Maintenance Center, Okayama, Japan; 7 Gastroenterology, Mabe Goryokaku Gastrointestinal Endoscopy Clinic, Hakodate, Japan; 8 Health Care Medicine, Kawasaki Medical School, Kawasaki Medical School, Japan

**Keywords:** Endoscopy Upper GI Tract, Diagnosis and imaging (inc chromoendoscopy, NBI, iSCAN, FICE, CLE), Precancerous conditions & cancerous lesions (displasia and cancer) stomach

## Abstract

**Background and study aims:**

Map-like redness is a known gastric mucosal finding observed after *Helicobacter pylori* eradication. Recently, we identified gastric lesions resembling barnacles and observed that they appear more commonly in cases of post- *H. pylori* infection. This study aimed to investigate clinicopathological characteristics of these barnacle-like lesions.

**Patients and methods:**

We analyzed clinical characteristics in 436 consecutive patients examined at Kawasaki Medical School General Medical Center. Histopathological examination was conducted in 43 patients, with 65 biopsies taken from barnacle-like lesions and 17 from the surrounding mucosa. The 43 patients comprised 20 patients biopsied at General Medical Center and 23 patients at Junpukai.

**Results:**

In total, 413 patients (208 women) were included in the analysis after excluding 23 patients based on exclusion criteria. Barnacle-like lesions were identified in 66 patients (16.0%), most frequently around the gastric angle, and were significantly more common in patients with mild atrophy of the fundic mucosa. Of the 66 patients with barnacle-like lesions, 65 were considered to have a post- *H. pylori* infection status and one was currently infected. Histopathological examination revealed intestinal metaplasia in 54 (83.1%) of the 65 biopsies from barnacle-like lesions. By contrast, all 17 biopsies from surrounding mucosa showed normal fundic mucosa without inflammation or atrophy.

**Conclusions:**

Barnacle-like lesions are a characteristic endoscopic finding of gastric mucosa post-infected with *H. pylori* and are histopathologically consistent with intestinal metaplasia.

## Introduction

In recent years, image enhancement systems such as narrow-band imaging (NBI), blue laser imaging (BLI), linked color imaging (LCI), and texture and color enhancement imaging have been developed 1 2 . *Helicobacter pylori* infection is a major cause of gastritis, peptic ulcers, and gastric cancer 3 4 5 6 , and then presents with various endoscopic findings through inflammation of the gastric mucosa. Endoscopic findings of *H. pylori* infection—uninfected, current infected, and post-infected—are published in the Kyoto Classification of Gastritis 7 . Yoshii et al. 8 assessed *H. pylori* infection endoscopically using the Kyoto Classification and reported the following sensitivity and specificity: 91.6% and 88.6% for uninfected patients, 75.0% and 89.9% for post-infected patients, and 59.5% and 94.7% for current infected patients, respectively. Studies have shown that *H. pylori* eradication can improve or eliminate endoscopic findings associated with *H. pylori* -induced gastritis, although a new endoscopic feature, termed map-like erythema, may appear 9 . This map-like erythema is characterized by multiple erythematous depressions in the gastric mucosa after *H. pylori* eradication and is histopathologically associated with intestinal metaplasia, a known risk factor for post-eradication gastric cancer 10 . In this study, we identified new small lesions resembling barnacles, referred to as “barnacle-like lesions” 11 , predominantly located around the gastric angle. Using advanced image enhancement systems, including LCI, BLI, and NBI, we describe this newly recognized type of gastric lesion and discuss its clinicopathological features.

## Patients and methods

### Study population

This study included 436 consecutive patients who underwent endoscopic examination at Kawasaki Medical School General Medical Center between January 1, 2015 and December 31, 2020. During this period, 17,360 upper gastrointestinal endoscopies were performed and 436 consecutive procedures conducted by K.H. were included in the study. Endoscopies were performed using a 5.9-mm diameter endoscope (EG-740 N; FUJIFILM Corporation, Saitama, Japan) or 5.8-mm diameter endoscope (GIF-XP290N; Olympus Medical Systems, Tokyo, Japan) equipped with LCI, BLI, or NBI systems. Of the 436 patients, 23 were excluded because of conditions preventing complete gastric observation, including postoperative stomach (10 patients), autoimmune gastritis (9 patients), eosinophilic gastritis (1 patient), advanced gastric cancer (2 patients), and food residue interference (1 patient).

### Definition and recognition of barnacle-like lesions

**Fig. 1:**
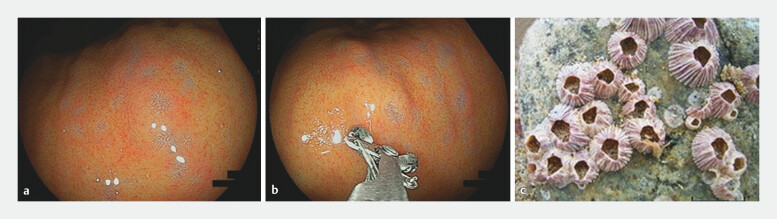
Morphological features of barnacle-like lesions. The barnacle-like lesions defined in this study, which when observed in close proximity, had **a** a slightly raised margin, a fine serrated morphology, and a depressed center; **b** were approximately 2 mm in size; and **c** resembled actual barnacles.

**Fig. 2:**
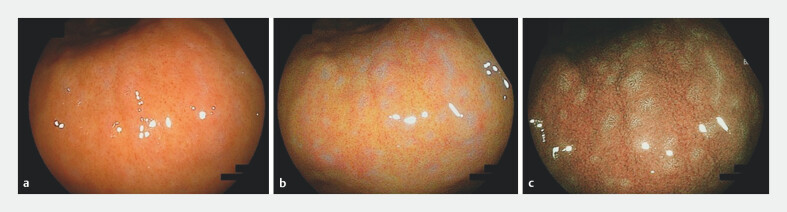
Visualization of barnacle-like lesions with image enhancement. **a** The lesions were difficult to visualize in white light. However, they became clearer with **b** LCI and **c** BLI enhancement.

Barnacle-like lesions were defined as small, raised lesions of approximately 2 mm in diameter with serrated edges and a central depression ( Fig. 1 **a,b** ). Although these lesions could be recognized under white-light observation, detailed morphological features required close-up imaging ( Fig. 2 **a-c** ). Lesions initially identified by K.H. were subsequently reviewed by three endoscopists: two specialists certified by the Japanese Society of Gastrointestinal Endoscopy (N.M. and T.M.) and one experienced endoscopist (A.S.). Cases with discordant interpretations were resolved by consensus.

### Data collection

Among the remaining 413 patients (208 women) with a mean age of 57.2 ± 13.0 years (range, 22–95), indications for endoscopy included gastric cancer screening (257 patients) and evaluation of digestive symptoms (156 patients). For each patient, presence and location of barnacle-like lesions, degree of gastric mucosal atrophy, and *H. pylori* infection status were assessed. *H. pylori* infection was categorized as current, uninfected, or post-infected based on endoscopic findings according to the Kyoto Classification of Gastritis 7 . In the First Edition of this classification, uninfected patients are characterized by absence of corpus atrophy, presence of regular arrangement of collecting venules (RAC) to the gastric angle, hematin adherence, and absence of diffuse erythema, mucosal swelling, intestinal metaplasia, and nodularity. Current infection is defined by presence of one or more of the following findings: corpus atrophy, diffuse erythema, mucosal swelling, cloudy mucus, spotting, loss of RAC, and nodularity. Post-infection is defined by presence of corpus atrophy and absence of diffuse erythema, mucosal swelling, and cloudy mucus, along with petechial erythema and presence of RAC in atrophic corpus mucosa. The stomach was anatomically divided into upper, middle, and lower regions, and mucosal atrophy was classified using the Kimura–Takemoto system as no atrophy (C1), closed atrophy (C2–C3), and open atrophy (O1–O3) 12 .

Analysis of frequency and clinical characteristics of barnacle-like lesions was conducted in the full consecutive population of 436 patients at Kawasaki Medical School General Medical Center. Histopathological examination of barnacle-like lesions was performed in 43 patients: 20 patients in whom biopsy was performed at Kawasaki Medical School General Medical Center and 23 patients in whom biopsies were obtained from barnacle-like lesion changes at Junpukai.

### Statistical analysis

Continuous variables with a normal distribution are typically reported as mean ± standard deviation, while those with a skewed distribution are reported as median (interquartile range). Student’s *t* -test was used to compare mean values of two independent groups. To compare categorical data, we used the chi-squared test with Yates’ correction or Fisher’s exact test.

### Ethics statement

This study adhered to the principles outlined in the Declaration of Helsinki and was approved by the Institutional Ethics Committee (approval no. 2021–0250, Junpukai 202190003). Informed consent was waived because the study was retrospective. Information regarding the study was made available through an opt-out notice on the facility website.

## Results

### Endoscopic findings and frequency of barnacle-like lesions

**Fig. 3:**
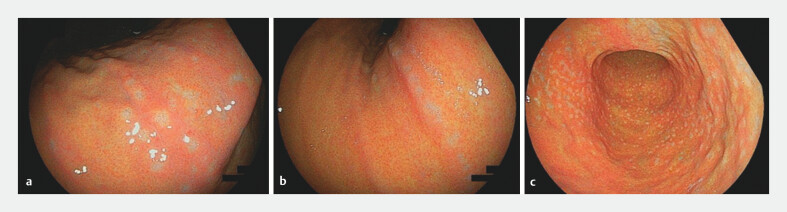
Distribution of barnacle-like lesions around the gastric angle. These lesions exhibited varied patterns, including **a** transverse, **b** longitudinal, and **c** diffuse distributions along the mucosal folds.

Barnacle-like lesions were identified in 66 of 413 (16.0%) consecutive endoscopies performed at the General Medical Center. These lesions were predominantly located in the middle region of the stomach (56 patients), with additional cases in the upper-middle region (10 patients). The lesions had a particular affinity for the gastric angle and the glandular border between the pyloric and fundic glands; none were found in the lower region. Each affected patient had more than 10 lesions. The distribution pattern varied, with some lesions arranged transversely or longitudinally along the mucosal folds and others showing a diffuse pattern ( Fig. 3 **a-c** ).

A comparison between patients with and without barnacle-like lesions showed no significant differences in sex or mean age. However, patients with these lesions exhibited significantly more closed gastric mucosal atrophy (mild atrophy) and a higher prevalence of *H. pylori* infection in the previously infected group than in the uninfected group ( Table 1 ). A history of oral proton pump inhibitor use was reported in six of 66 patients with barnacle-like lesions and in 51 of 347 patients without such lesions, with no statistically significant difference ( *P* = 0.329).

**Table:** **Table 1** Clinicopathological features of patients with and without barnacle-like lesions.

	Barnacle-like lesion (+)	Barnacle-like lesion (−)	*P* value
Patients	66	347	
Male/female	31/35	174/173	0.69
Age, years	60.2 ± 12.2	56.7 ± 13.1	0.055
Grade of gastric atrophy			
C1 (no atrophy)	0	221	< 0.001 ^*^
Mild (closed, C2+C3)	53	74	< 0.001 ^†^
Severe (open, O1+O2+O3)	13	52	
*H. pylori* infection			
Uninfected	0	209	< 0.001 ^‡^
Previous	65	107	
Current	1	31	
Data are presented as n or mean ± standard deviation. ^*^ C1 vs. other grades of atrophy. ^†^ Mild (closed) vs. severe (open) atrophy. ^‡^ Uninfected vs. other infection status.			

Because map-like redness is a known finding in post-infected patients, we examined 172 of 413 patients diagnosed as post-infected. Among them, 65 were in the barnacle-like lesion group and 107 in the non-lesion group. Map-like redness was present in two patients with barnacle-like lesions and in nine patients without them, showing no statistically significant difference ( *P* = 0.211) ( Table 2 ).

**Table:** **Table 2** Relationship between map-like erythema and barnacle-like lesions.

	Map-like erythema (+) n = 11	Map-like erythema (−) n = 161
Barnacle-like lesions (+), n = 65	2	63
Barnacle-like lesions (−), n = 107	9	98

### Histopathological examination of barnacle-like lesions

**Fig. 4:**
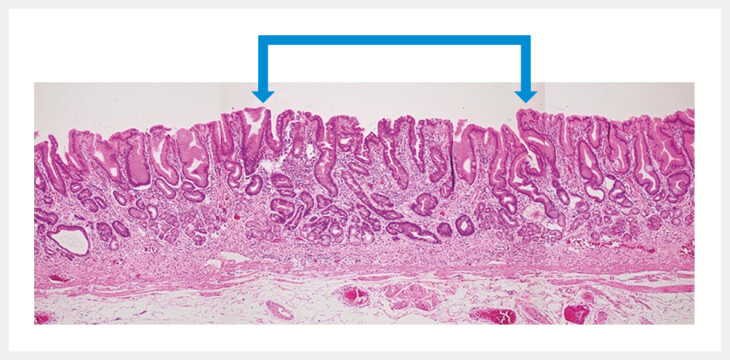
Histopathological image of a barnacle-like lesion. The central depression, marked by arrowheads, represents intestinal metaplasia and is surrounded by non-atrophic fundic gastric mucosa. Moderate inflammation is present in the central intestinal metaplasia.

**Fig. 5:**
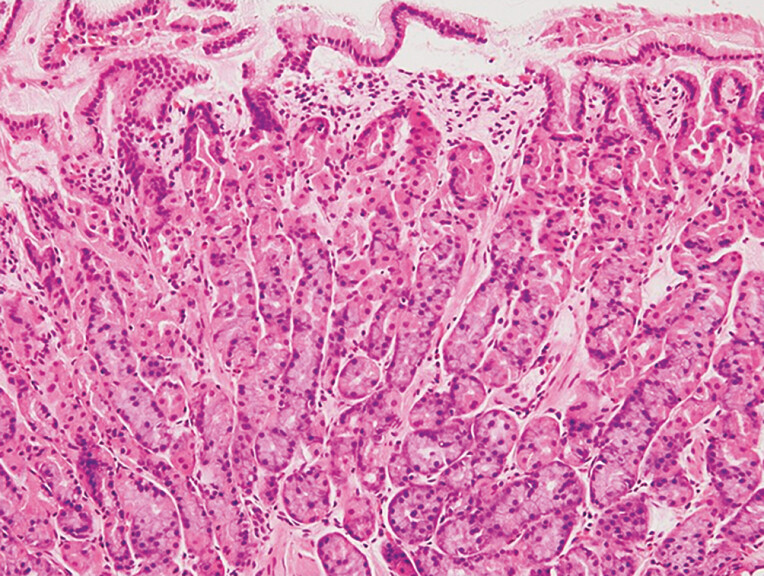
Biopsy specimen from the surrounding mucosa of a barnacle-like lesion. The specimen showed gastric fundic gland mucosa without atrophy or inflammation.

Histopathological analysis was performed on gastric biopsy samples of the barnacle-like lesions, encompassing 65 lesions from 43 patients from the General Medical Center (20 patients) and Junpukai (23 patients). The primary histopathological feature observed in 54 (83.1%) of these 65 lesions was a slightly depressed area of intestinal metaplasia surrounded by gastric fundic gland tissue ( Fig. 4 ). In the 54 lesions exhibiting intestinal metaplasia, severity of inflammation was graded using the updated Sydney system 13 : 13 (24.1%) lesions showed no inflammation, 25 (46.3%) showed mild inflammation, 12 (22.2%) showed moderate inflammation, and 4 (7.4%) showed severe inflammation ( Table 3 ). For the 17 biopsy samples obtained from mucosa surrounding the barnacle-like lesions, histopathological examination revealed normal gastric fundic mucosa without evidence of atrophy or inflammation ( Fig. 5 ).

**Table:** **Table 3** Histopathological features of barnacle-like lesions.

Intestinal metaplasia		
Positive	54/65	(83.1%)
Negative	11/65	(16.9%)
Grade of inflammation in the intestinal metaplasia		
None	13/54	(24.1%)
Mild	25/54	(46.3%)
Moderate	12/54	(22.2%)
Severe	4/54	(7.4%)

Among the reviewed cases, three patients at the General Medical Center had a history of endoscopic treatment for early-stage well-differentiated adenocarcinoma, whereas two of the 23 patients at Junpukai had early-stage well-differentiated adenocarcinoma.

## Discussion

This study identified a novel type of gastric lesion, referred to as a barnacle-like lesion, using advanced image enhancement systems including LCI, BLI, and NBI. Although visible under white light, detailed morphology of these lesions was challenging to discern without image enhancement. Barnacle-like lesions were significantly more prevalent among patients with previous *H. pylori* infection, particularly around the gastric angle.

The preferred location for barnacle-like lesions was the gastric fundic mucosa, extending from the gastric angle to the antrum. Histopathologically, the central depression of each lesion was characterized by intestinal metaplasia with surrounding normal gastric fundic gland mucosa. Endoscopic evaluations revealed that 80.3% of patients (53/66) with barnacle-like lesions had mild mucosal atrophy (classified as C2 or C3) in the gastric fundus, suggesting that these lesions commonly occur in areas with mild atrophy near the boundary between the pyloric and fundic glands. This pattern indicates that barnacle-like lesions may be a post-infection feature observed in mildly atrophic mucosa along the gastric fundus–pyloric gland border.

Previous studies have revealed map-like redness as another feature of post-eradication, typically presenting as large, depressed areas of intestinal metaplasia 9 10 . By contrast, barnacle-like lesions, which manifest as smaller, dotted areas of intestinal metaplasia, were found in only 2 of 66 patients with map-like lesions, indicating a distinct presentation. Given that one case of barnacle-like lesions was observed in an actively infected patient, it is possible that some of these lesions preexisted but became more prominent after eradication. The mechanism by which barnacle-like lesions are observed in post-infected patients is thought to be that *H. pylori* infection causes inflammation, edema, and epithelial hyperplastic changes in the gastric mucosa, which mask the intestinal metaplasia, and that *H. pylori* eradication improves inflammation, edema, and epithelial hyperplastic changes, whereas the intestinal metaplasia remains and becomes more evident. Among 32 patients diagnosed as currently infected, one (3.13%) had barnacle-like lesions; none were found in 209 uninfected patients, whereas 65 (37.8%) of 172 post-infected patients exhibited barnacle-like lesions. This suggests that such lesions may serve as an endoscopic marker of past *H. pylori* infection because their presence was significantly more common in post-infected individuals.

Histopathological examination revealed that the barnacle-like lesions primarily consisted of depressed intestinal metaplasia surrounded by gastric fundus glands, with mild to moderate inflammatory cell infiltration. Because intestinal metaplasia is a known risk factor for differentiated adenocarcinoma 14 , the potential relationship between barnacle-like lesions and gastric cancer warrants further investigation. Kanzaki et al. 15 diagnosed intestinal metaplasia of the corpus based on endoscopic findings and explored its progression. Marcos et al. 16 assessed the extent of intestinal metaplasia endoscopically and reported a high correlation with histopathological findings, using this approach as a screening tool for early gastric neoplasia. These studies utilized magnifying endoscopy with NBI. In contrast, the barnacle-like lesions described in the present study were visible with normal magnification using image enhancement techniques. Because of the small number of gastric cancer cases in this study, we were unable to investigate the association between barnacle-like lesions and gastric cancer. However, if a link can be established through studies involving a larger number of cases, these lesions may prove to be a simple and useful marker for assessing gastric cancer risk.

A key limitation of this study is its single-center, retrospective design and the involvement of only one endoscopist, which may affect generalizability of the findings. In addition, the relatively small number of patients with gastric cancer limits the ability to establish a clear relationship between barnacle-like lesions and cancer risk.

## Conclusions

In conclusion, presence of barnacle-like lesions on endoscopy is likely indicative of post- *H. pylori* infection in the gastric mucosa, and histopathologically, these lesions correspond to intestinal metaplasia. Further prospective studies involving larger patient populations are needed to clarify the potential association between barnacle-like lesions and gastric cancer.
