# Correction: Barnacle-like lesions in the gastric mucosa: Clinicopathological study of a novel endoscopic finding

**DOI:** 10.1055/a-2703-2842

**Published:** 2025-09-19

**Authors:** Aya Sunago, Takahisa Murao, Ken Haruma, Maki Ayaki, Noriaki Manabe, Minoru Fujita, Takashi Akiyama, Mitsuhiko Suehiro, Hirofumi Kawamoto, Kazuhiko Inoue, Katsuhiro Mabe, Eiichiro Kanda, Tomoari Kamada

**Affiliations:** 1 Health Care Medicine, Kawasaki Medical School General Medical Center, Okayama, Japan; 2 12864 General Internal Medicine 2, Kawasaki Medical School, Kurashiki, Japan; 3 Gastroenterology and Hepatology, HITO Medical Center, Shikokuchuou, Japan; 4 12864 Division of Endoscopy and Ultrasonography, Department of Clinical Pathology and Laboratory Medicine, Kawasaki Medical School, Kurashiki, Japan; 5 Pathology, Kawasaki Medical School General Medical Center, Okayama, Japan; 6 Health Care Medicine, Junpukai Health Maintenance Center, Okayama, Japan; 7 Gastroenterology, Mabe Goryokaku Gastrointestinal Endoscopy Clinic, Hakodate, Japan; 8 Health Care Medicine, Kawasaki Medical School, Kawasaki Medical School, Japan



In the above-mentioned article Table 1 was corrected.

**Table:** **Table 1** Clinicopathological features of patients with and without barnacle-like lesions.

	Barnacle-like lesion (+)	Barnacle-like lesion (−)	*P* value
Patients	66	347	
Male/female	31/35	174/173	0.69
Age, years	60.2 ± 12.2	56.7 ± 13.1	0.055
Grade of gastric atrophy			
C1 (no atrophy)	0	221	< 0.001 ^*^
Mild (closed, C2+C3)	53	74	< 0.001 ^†^
Severe (open, O1+O2+O3)	13	52	
*H. pylori* infection			
Uninfected	0	209	< 0.001 ^‡^
Previous	65	107	
Current	1	31	
Data are presented as n or mean ± standard deviation. ^*^ C1 vs. other grades of atrophy. ^†^ Mild (closed) vs. severe (open) atrophy. ^‡^ Uninfected vs. other infection status.			

This was corrected in the online version on 19.09.2025.

